# The efficacy of lactobacillus reuteri in conjunction with racecadotril in managing pediatric rotavirus enteritis and its impact on intestinal mucosa and immune function

**DOI:** 10.4314/ahs.v25i2.6

**Published:** 2025-06

**Authors:** Zhihua He, Lin Lan, Nini Chen, Yanling Cheng, Sitang Gong

**Affiliations:** Digestive Laboratory, Department of Gastroenterology, Guangzhou Women and Children's Medical Center, Guangzhou Medical University, Guangzhou 510623, China

**Keywords:** Lactobacillus reuteri, Racecadotril, Pediatric rotavirus enteritis, Intestinal microbiota, Intestinal mucosal barrier function, Immune function

## Abstract

**Background:**

This study aimed to evaluate the effectiveness of combining Lactobacillus reuteri with racecadotril in treating pediatric rotavirus enteritis, focusing on its impact on intestinal mucosa and immune function.

**Methodology:**

Eighty-five children diagnosed with pediatric rotavirus enteritis were randomly divided into two groups: the control group (43 cases) receiving racecadotril alone and the observation group (42 cases) treated with both Lactobacillus reuteri and racecadotril. Evaluation of the RV conversion rate and treatment efficacy was conducted at 3d, 5d, and 7d post-treatment. The study also assessed changes in intestinal mucosal barrier function, immune response, and intestinal microbiota.

**Results:**

The observation group exhibited significantly higher RV conversion rates at 3d, 5d, and 7d post-treatment compared to the control group (P<0.05), reaching 61.90%, 76.19%, and 92.86%, respectively. Following treatment, the observation group showed improvements in mucosal barrier function, increased CD4+ levels, decreased AGEs, D-lactic acid, endotoxins, and CD8+ levels. There were notable changes in intestinal microbiota, with elevated Lactobacillus and Bifidobacterium levels and decreased Escherichia coli.

**Conclusions:**

Combining Lactobacillus reuteri and racecadotril in treating pediatric rotavirus enteritis demonstrated efficacy in regulating intestinal microbiota, alleviating mucosal barrier dysfunction, improving immune function, and enhancing treatment effectiveness.

## Introduction

Rotavirus enteritis, a prevalent gastrointestinal disease in children, is characterized by its acute onset, short duration, and high infectivity.^1^ Despite a global reduction in incidence rates attributed to the widespread use of rotavirus vaccines, the disease continues to impose a significant health burden in specific regions and among certain populations, particularly in areas with limited access to vaccination programs or where vaccine efficacy is reduced due to various factors such as malnutrition and immune deficiencies.^2^ Conventional treatment methods prioritize symptomatic relief by employing antidiarrheal agents like racecadotril, but fail to address the underlying issue of regulating the intestinal environment and achieving a restored, balanced intestinal ecology.^3^ However, due to the fact that the intestine is the second largest immune organ in the human body, the intestinal microbiota and intestinal mucosal barrier play a crucial role in maintaining overall health.^4^ Hence, placing emphasis on the restoration and preservation of a balanced intestinal microbiota and fortifying barrier function is vital for the successful treatment and prevention of recurrent pediatric rotavirus enteritis. Lactobacillus reuteri, as a natural probiotic, has been substantiated for its capacity in effectively suppressing the growth of various fungi and viruses while exerting a beneficial regulatory influence on human immune function.^5^ This study aims to assess the efficacy of a combined therapy involving Lactobacillus reuteri and racecadotril in the treatment of pediatric rotavirus enteritis. Additionally, we will investigate the effects of this combination therapy on intestinal mucosal health and immune function. Through rigorous clinical evaluations and laboratory analyses, we seek to provide valuable insights into the potential benefits of this treatment regimen. This research contributes to a more comprehensive understanding of probiotic and antidiarrheal treatments, addressing the specific needs of pediatric patients with rotavirus enteritis.

## Materials and Methods

### General Information

A total of 85 children diagnosed with pediatric rotavirus enteritis were enrolled and randomly allocated into two groups: the control group (43 cases) and the observation group (42 cases). Treatment with racecadotril was administered to the control group, which consisted of children aged between 3 months and 6 years, with an average age of 2.41±0.67 years. Among the participants, there were 27 boys and 16 girls, experiencing a daily frequency of 6-11 episodes of diarrhea, with an average of 8.21±2.16 episodes. Furthermore, 31 children had fever, and 38 children experienced vomiting. For the observation group, treatment involved a combined approach using Lactobacillus reuteri and racecadotril, with participants ranging in age from 3 months to 6 years and having an average age of 2.35±0.74 years. The group comprised 24 boys and 18 girls, encountering a daily frequency of 6-10 episodes of diarrhea, averaging 8.25±2.21 episodes. Moreover, among these children, 30 had fever and 39 had episodes of vomiting. Comparison of the general characteristics between the two groups revealed no significant disparities (P>0.05). This study was approved by the ethics committee of Guangzhou Women and Children's Medical Center(Approval number: 20200532-LL). Signed written informed consents were obtained from the patients and/or guardians.

Inclusion criteria for the study encompassed the following: (1) Participants aged between 3 months and 6 years: This age range was chosen because it represents the population most vulnerable to acute infectious diarrhea caused by rotavirus (RV) infection. Young children, especially those under the age of 6, are more susceptible to severe symptoms and complications from RV infections due to their developing immune systems and limited previous exposure to the virus.; (2) Meeting the criteria outlined in the “Clinical Practice Guidelines for Acute Infectious Diarrhea in Chinese Children”, 6 which include rapid onset with vomiting preceding diarrhea, often associated with respiratory tract infections and electrolyte abnormalities, along with positive laboratory test results for rotavirus (RV) antigen; (3) No administration of antibiotics following the onset of the disease; (4) Obtaining written informed consent from the immediate relatives of the children.

The study specified the following exclusion criteria: (1) Severe malnutrition or dehydration; (2) Chronic diarrhea; (3) Organic gastrointestinal conditions; (4) Acute diarrhea attributed to other pathogens; (5) Recent use of probiotics within the last 3 months; (6) Congenital abnormalities affecting intestinal development; (7) Allergic predisposition.

## Methods

Treatment for the control group involved the oral administration of racecadotril tablets (Yangzi River Pharmaceutical Group, Beijing Haiyan Pharmaceutical Co., Ltd., Beijing, China). The recommended dosage was 1.5 mg per kilogram of body weight, administered three times daily, for a continuous duration of 7 days. Treatment for the observation group consisted of a combination approach involving the oral administration of Lactobacillus reuteri (Swedish Biotechnology Company) and racecadotril. Lactobacillus reuteri was administered daily at a dosage of 1×10^8 CFU for a period of 7 days. The administration method and dosage of racecadotril were analogous to those employed in the control group.

Racecadotril is a medication with well-established pharmacological action. It functions as an enkephalinase inhibitor, specifically targeting the enzyme that degrades endogenous enkephalins in the gastrointestinal tract. By inhibiting enkephalin degradation, racecadotril enhances the local action of enkephalins, which serve as natural intestinal antisecretory agents. This action leads to reduced fluid secretion in the intestines, effectively alleviating diarrhea symptoms in patients with rotavirus enteritis.

Lactobacillus reuteri, on the other hand, operates through its probiotic properties. It colonizes the intestinal tract and plays a crucial role in modulating the gut microbiota composition. Additionally, it promotes the strengthening of the intestinal mucosal barrier, reducing the permeability to pathogens and toxins. Furthermore, Lactobacillus reuteri is known for its immune-modulating effects, enhancing the host's immune response against pathogens such as rotavirus.

### Outcome Measures

#### RV Conversion

The presence of rotavirus-specific antigen in fecal samples was assessed using enzyme-linked immunosorbent assay (ELISA), with monitoring continued until a negative result was obtained.

#### Intestinal Mucosal Barrier Function

Patients had their peripheral venous blood (5 ml) collected both before treatment and after 7 days. The collected blood samples were then centrifuged at a radius of 10 cm with a speed of 3000 r/min for a duration of 10 min. Afterward, the supernatant was collected for assessing the levels of AGEs, D-lactic acid, endotoxins, and diamine oxidase (DAO) employing enzyme-linked immunosorbent assay (ELISA).

#### Immune Function

Serum samples were obtained and utilized to measure the levels of CD4+, CD8+, and CD4+/CD8+ through immunofluorescence assay. The immunofluorescence reagent kits employed in this study were sourced from Corning, a reputable US company. These markers are crucial for understanding the immune system's performance. CD4+ T cells play a key role in coordinating immune responses, while CD8+ T cells directly target and eliminate infected cells. The CD4+/CD8+ ratio reflects the balance between these cell types. By measuring these parameters, we can evaluate an individual's immune system health and its ability to respond to threats like infections, providing insights into overall immune system function.

#### Intestinal Microbiota

Prior to treatment commencement and after 7 days of treatment, stool samples were collected from patients. Subsequently, microbial culture and quantification were carried out on these samples. The presence of bacterial DNA in the stool samples was detected using the Wang RF method. Furthermore, fluorescence techniques were employed to quantitatively determine the quantities of Escherichia coli, Lactobacillus, Bifidobacterium, and Enterococcus present in the samples.

#### Efficacy Criteria

Marked effectiveness: Normal stool appearance and frequency within 72 hours, disappearance of accompanying symptoms such as vomiting and fever; Effectiveness: Improvement in stool appearance and frequency within 72 hours, with symptom reduction; Ineffectiveness: No improvement or worsening within 72 hours.

### Statistical Analysis

Utilizing Statistic Package for Social Science (SPSS) 26.0 (IBM, Armonk, NY, USA), the data were subjected to analysis. Descriptive statistics (mean ± standard deviation) were employed to summarize normally distributed and homogeneous data, encompassing daily frequency of diarrhea, endotoxins, and D-lactic acid. Comparisons between groups were conducted using Student's t-test and chi-square test, with a threshold of P<0.05 denoting statistical significance.

## Results

### RV Conversion Rate

The RV conversion rates in the observation group after 3 days, 5 days, and 7 days of treatment reached 61.90%, 76.19%, and 92.86%, respectively. Notably, these rates were considerably higher than those observed in the control group (39.53%, 55.81%, and 72.09%). Comparison of the RV conversion rates between the two groups, conducted using a chi-squared test, revealed no significant disparities (P<0.05) (Table 1).

**Table 1:** RV conversion rate (%)

Group	n	3d after	5d after	7d after
Control	43	17 (39.53)	24 (55.81)	31 (72.09)
Observation	42	26 (61.90)	32 (76.19)	39 (92.86)
*χ* ^2^		4.254	3.925	6.303
*P*		0.039	0.048	0.012

### Intestinal Mucosal Barrier Function

Before treatment, there was no significant difference in intestinal mucosal barrier function between the two groups (P>0.05). After comparing before and after treatment, both groups showed significant reductions in AGEs, D-lactic acid, endotoxins, and DAO. The observation group had lower levels of AGEs, D-lactic acid, endotoxins, and DAO compared to the control group,as determined by a t-test (P<0.05) (Table 2).

**Table 2:** Intestinal mucosal barrier function

Group	n	AGEs (ng/L)		D-lactic acid (mg/L)		endotoxins (EU/mL)		DAO (U/L)	

		Before	After	Before	After	Before	After	Before	After
Control	43	421.25±82.26	235.23±74.05*	4.79±0.52	2.87±0.41*	2.08±0.67	0.81±0.52*	2.75±0.37	1.61±0.34*
Observation	42	415.69±94.73	165.89±60.27*	4.68±0.61	2.02±0.35*	2.14±0.64	0.37±0.26*	2.81±0.40	0.98±0.31*
*t*		0.289	4.728	0.895	7.305	0.422	4.916	0.718	8.921
*P*		0.773	0.000	0.373	0.000	0.674	0.000	0.475	0.000

Compared with prior to treatment

* P<0.05

### Immune Function

Before treatment, there was no significant difference in immune function between the two groups (P>0.05). After comparing before and after treatment, the observation group showed significant increases in CD4+ and CD4+/CD8+ levels, as well as a significant decrease in CD8+ levels. There was no significant difference in immune function in the control group compared to before treatment (P>0.05). The observation group had higher levels of CD4+ and CD4+/CD8+ and lower levels of CD8+ compared to the control group,as determined by a t-test (P<0.05) (Table 3).

**Table 3:** Immune function

Group	n	CD4+		CD8+		CD4+/CD8+	

		Before	After	Before	After	Before	After
Control	43	30.25±3.57	31.04±3.91	32.85±3.11	31.56±2.98	0.92±0.27	0.98±0.32
Observation	42	30.14±3.63	38.74±4.02*	32.96±2.97	25.21±2.26*	0.91±0.31	1.52±0.38*
*t*		0.141	6.627	0.167	5.394	0.159	7.093
*P*		0.888	0.000	0.868	0.000	0.874	0.000

Compared with prior to treatment

* P<0.05

### Intestinal Microbiota

Before treatment, there was no significant difference in intestinal microbiota between the two groups (P>0.05). After comparing before and after treatment, the observation group showed significant increases in Lactobacillus and Bifidobacterium, and a significant decrease in Escherichia coli, while Enterococcus showed no significant difference compared to before treatment (P>0.05). In the control group, there was no significant difference in the intestinal microbiota after treatment compared to before treatment (P>0.05). The observation group had higher levels of Lactobacillus and Bifidobacterium and lower levels of Escherichia coli compared to the control group, with no significant difference in Enterococcus compared to the control group,as determined by a t-test (P>0.05) (Table 4).

**Table 4:** Intestinal flora (log10^n^CFU/g)

Group	n	Lactobacillus		Bifidobacterium		Escherichia coli		Enterococcus	

		Before	After	Before	After	Before	After	Before	After
Control	43	6.25±1.41	6.54±1.52	5.58±1.30	5.84±1.41	6.58±1.05	6.51±1.24	7.55±0.69	7.44±0.89
Observation	42	6.14±1.36	8.36±1.49*	5.51±1.35	9.75±1.56*	6.49±1.11	6.48±1.17	7.60±0.75	6.05±1.02*
*t*		0.366	5.573	0.244	12.129	0.384	0.115	0.320	6.699
*P*		0.715	0.000	0.808	0.000	0.702	0.909	0.750	0.000

Compared with prior to treatment

* P<0.05

### Clinical Efficacy

The total effective rate in the observation group was 92.86% (24 cases of marked effectiveness and 15 cases of effectiveness), which was significantly higher than the rate in the control group (76.74%, 15 cases of marked effectiveness and 18 cases of effectiveness) with statistical significance, conducted using a chi-squared test, (P<0.05) (Table 5).

**Table 5:** Clinical efficacy (%)

Group	n	Marked effectiveness	Effectiveness	Ineffectiveness	Overall rate
Control	43	15 (34.88)	18 (41.86)	10 (23.26)	33 (76.74)
Observation	42	24 (57.14)	15 (35.71)	3 (7.14)	39 (92.86)
*χ* ^2^					4.258
*P*					0.039

### Comparison of Safety

In this study, a comprehensive assessment of safety was conducted to ensure the well-being of the pediatric patients. Notably, no adverse effects were observed during the course of the treatment. Adverse effects were monitored through regular clinical evaluations, including physical examinations and laboratory tests, to detect any potential side effects or complications. Furthermore, caregivers and parents were encouraged to report any unusual symptoms or concerns, and their feedback was actively solicited throughout the study period. This proactive approach to monitoring adverse effects contributed to the overall safety profile of the combined treatment regimen. No adverse reactions were observed in either group.

## Discussion

Rotavirus enteritis, commonly known as rotavirus gastroenteritis, is a common gastrointestinal disease in children caused by rotavirus infection, which is mainly manifested as symptoms such as diarrhea, vomiting, and fever.^7^ Rotavirus primarily attacks intestinal epithelial cells, leading to damage to the intestinal mucosa and decreased absorption function, thereby triggering diarrhea.^8^ In addition, viral invasion also stimulates the body's immune response and produces an inflammatory response, and excessive inflammation can further damage the intestinal mucosa and affect intestinal immune function.^9^ Currently, levcarnitine is widely used as an antidiarrheal drug in the treatment of rotavirus enteritis. Although it can effectively alleviate diarrhea symptoms, its main effect is symptomatic treatment and cannot comprehensively address problems such as intestinal dysbiosis, intestinal mucosal damage, and immune dysfunction.^10^ In recent years, the positive effects of probiotics in maintaining gut microbiota stability, repairing intestinal mucosa, and regulating immune function have been gradually recognized.^11^ Lactobacillus rhamnosus, belonging to lactic acid bacteria, can not only regulate gut microbiota and reduce the growth of harmful bacteria but also enhance intestinal mucosal barrier and strengthen the body's immune response, thereby achieving a more comprehensive therapeutic effect.^12^ In this study, we explored the mechanism of action of Lactobacillus rhamnosus in the context of treating rotavirus enteritis in children. Lactobacillus rhamnosus is known for its probiotic properties, including its ability to modulate the intestinal microbiota and enhance the mucosal barrier function. In the context of rotavirus enteritis, these mechanisms take on particular relevance. Specifically, Lactobacillus rhamnosus has been shown to exert anti-inflammatory effects within the gastrointestinal tract, reducing the severity of intestinal inflammation often associated with rotavirus infection. Furthermore, its ability to enhance the integrity of the mucosal barrier is crucial in preventing the invasion and replication of rotavirus in the intestinal epithelium. Moreover, Lactobacillus rhamnosus can also support the host's immune response, which is especially significant in pediatric patients with rotavirus enteritis. By promoting the production of immunoglobulins and enhancing immune cell activity, it contributes to a more robust defense against rotavirus, aiding in both recovery and prevention of reinfection. In summary, the mechanism of action of Lactobacillus rhamnosus aligns closely with the goals of our study, where we aimed to provide a more effective treatment strategy for rotavirus enteritis in children. Its anti-inflammatory, mucosal barrier-enhancing, and immune-modulating properties make it a valuable component of the combined treatment regimen, addressing the specific needs of pediatric patients with this condition.^12^ Therefore, the incorporation of both levocarnitine and Lactobacillus rhamnosus in treating regimen holds promise as a more comprehensive and effective approach for addressing rotavirus enteritis.

The results of this study showed that the RV conversion rate in the observation group at 3 d, 5 d, and 7 d was higher than that in the antidiarrheal group. This indicates that compared to the single use of levocarnitine, the treatment method of adding Lactobacillus rhamnosus is more effective in a shorter period of time. Specifically, levels of advanced glycation end-products (AGEs), D-lactic acid, endotoxin, and DAO in the observation group were significantly reduced, while the levels of CD4+ and CD4+/CD8+ were significantly increased. Meanwhile, the levels of Lactobacillus and Bifidobacterium in the observation group after treatment were higher than those in the antidiarrheal group, while Escherichia coli was lower than the antidiarrheal group. This indicates that the observation group has improved intestinal mucosal function and immune function and helps maintain and restore a healthy gut microbiota balance. The reason for this analysis is that Lactobacillus rhamnosus is a probiotic, and one of its main metabolites is rhamnosylceramide, a special metabolite with significant antibacterial effects that can inhibit the growth of various harmful bacteria sch as Escherichia coli and Salmonella, thus maintaining and stabilizing the gut's microbial environment.^13^,^14^ In addition, Lactobacillus rhamnosus can increase the concentration of butyric acid in the intestines. Butyric acid, as a short-chain fatty acid, provides energy to intestinal epithelial cells and further promotes their proliferation and repair, playing a crucial role in the regeneration and maintenance of intestinal epithelial tissue.^15^ Furthermore, this strain of lactobacillus can produce various beneficial enzymes in the intestines, such as lipase and bile salt hydrolase, which help optimize the intestinal pH environment, inhibit the growth of harmful bacteria, and improve the digestion and absorption rate of food.^16^ Additionally, rhamnosylceramide can induce oxidative stress in pathogens, as the imbalance of intracellular free radicals and reactive oxygen species (ROS) levels restricts the survival and reproduction of pathogens.^16^ Moreover, rhamnosylceramide can activate CD4+ cells in the body, thereby recognizing host cells infected by pathogens and initiating an immune response to eliminate pathogens.^17^ Finally, this medication can induce the production of specific immunoglobulins such as IgG and IgA, which act to neutralize and recognize invading pathogens in humoral immune responses. Therefore, through the mechanisms mentioned above, rhamnosylceramide can not only directly inhibit pathogens but also enhance the body's immune response, thereby more effectively treating rotavirus enteritis.^18^

This study further analyzed the overall efficacy and the results showed that the observation group had a total effective rate of 92.86%, significantly higher than the antidiarrheal group with a rate of 76.74%. This further confirms the important role of Lactobacillus rhamnosus in the treatment of pediatric rotavirus enteritis. This is because the combined use of levocarnitine and Lactobacillus rhamnosus helps maintain normal intestinal motility, ensures the balance of moisture and electrolytes, and effectively alleviates diarrhea symptoms.^19^ In addition, the balance of gut microbiota has a direct impact on the secretion of gastrointestinal hormones, and this combined effect can also lead to a decrease in the level of gastrointestinal hormones, further stabilizing the intestinal environment.^20^ Therefore, the synergistic effect of Lactobacillus rhamnosus and levocarnitine is of great significance in maintaining gut health and preventing intestinal-related diseases such as diarrhea. Furthermore, no adverse reactions occurred in both groups in this study, indicating that the combined treatment approach is relatively safe and can be clinically promoted. However, it is important to acknowledge the limitations of our study. Firstly, the study sample size was relatively small, and the study was conducted in a single-center setting. This may limit the generalizability of our findings to a broader population. Secondly, the follow-up period was relatively short, and the long-term effects of this combination therapy remain to be explored. Additionally, while efforts were made to minimize bias, the potential for selection bias in patient enrollment cannot be entirely ruled out.

## Conclusion

The utilization of Lactobacillus rhamnosus in conjunction with levocarnitine proves to be more effective than the singular administration of levocarnitine in treating pediatric rotavirus enteritis. Not only does it increase the RV conversion rate, but it also helps improve intestinal mucosal barrier function, immune function, and regulate gut microbiota, thereby increasing the overall efficacy of the treatment.
